# The *Drosophila* Larval Locomotor Circuit Provides a Model to Understand Neural Circuit Development and Function

**DOI:** 10.3389/fncir.2021.684969

**Published:** 2021-07-01

**Authors:** Iain Hunter, Bramwell Coulson, Aref Arzan Zarin, Richard A. Baines

**Affiliations:** ^1^Division of Neuroscience and Experimental Psychology, Faculty of Biology, Medicine and Health, Manchester Academic Health Science Centre, School of Biological Sciences, University of Manchester, Manchester, United Kingdom; ^2^Department of Biology, The Texas A&M Institute for Neuroscience, Texas A&M University, College Station, TX, United States

**Keywords:** *Drosophila*, circuit, connectome, locomotion, critical period, variability

## Abstract

It is difficult to answer important questions in neuroscience, such as: “how do neural circuits generate behaviour?,” because research is limited by the complexity and inaccessibility of the mammalian nervous system. Invertebrate model organisms offer simpler networks that are easier to manipulate. As a result, much of what we know about the development of neural circuits is derived from work in crustaceans, nematode worms and arguably most of all, the fruit fly, *Drosophila melanogaster*. This review aims to demonstrate the utility of the *Drosophila* larval locomotor network as a model circuit, to those who do not usually use the fly in their work. This utility is explored first by discussion of the relatively complete connectome associated with one identified interneuron of the locomotor circuit, A27h, and relating it to similar circuits in mammals. Next, it is developed by examining its application to study two important areas of neuroscience research: critical periods of development and interindividual variability in neural circuits. In summary, this article highlights the potential to use the larval locomotor network as a “generic” model circuit, to provide insight into mammalian circuit development and function.

## Introduction

**FIGURE 1:**
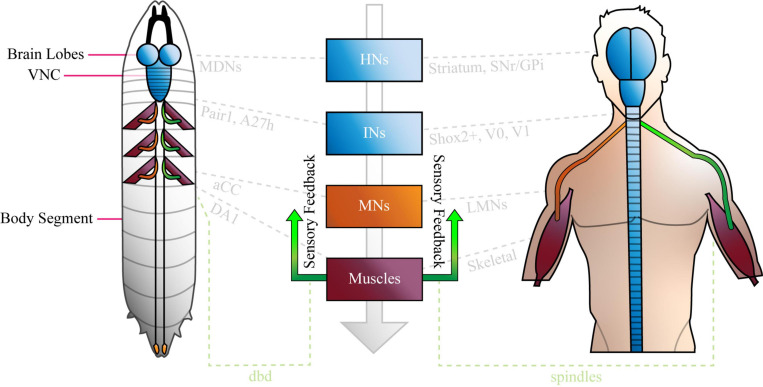
The Basic Anatomy of the Anatomy of the Nervous System is conserved between *Drosophila* Larvae and Humans. The *Drosophila* larval nervous system is comprised of a brain (two lobes) populated by higher neurons, a ventral nerve cord (VNC) of interneurons, plus sensory inputs (green) from, and motor outputs (orange) to the periphery. The VNC is segmented, and each compartment sends nerves via central tracts, to corresponding body segments. Only three segments are shown with nerves and muscles for clarity. The human nervous system is similar, in that it features a bi-lobed brain of higher neurons, and a spinal cord of interneurons and motor neurons that is analogous to the larval VNC. Like the VNC, the spinal cord is segmented. Sensory inputs (green) and motor outputs (orange), travel to and from it, respectively. Note that both panels were designed to represent the overall organization of each nervous system simply, and not for precise anatomical correctness. Shared cell types are shown in central boxes, and examples of similar, specific brain regions, neurons and muscles are given in grey. HNs, higher neurons; INs, interneurons; MNs, motor neurons; SNr, basal ganglia; GPi, globus pallidus interna; aCC, anterior corner cell; LMNs, lower motor neurons; dbd, dorsal bipolar dendrite neuron. See “Comparison of *Drosophila* and Mammalian Neural Networks” for a more detailed comparison of the two nervous systems.

Relatively little is known about how neural networks develop and function, and one factor that has impacted progress in this field, is the complexity and inaccessibility of the mammalian nervous system. The human nervous system (NS) is a vast network of tens of billions of neurons connected by trillions of synapses (Azevedo et al., 2009), and ethical and practical considerations prevent direct experimentation on them. Research conducted on human circuits has, therefore, traditionally been constrained to relatively low resolution, non-invasive techniques such as functional magnetic resonance imaging, computerized axial tomography and electromyography. Our understanding of the (human) NS is correspondingly restricted to regions of the brain (e.g., motor cortex) and categories of neuron in the peripheral nervous system (e.g., IaIN interneurons and IIb motor neurons). Recent work shows that we can study murine and other model vertebrate nervous systems in more detail (Kiehn, 2016; Arber and Costa, 2018; Grillner and El Manira, 2020), however, the field lacks the power to describe the roles of individual neurons in mammalian circuits on any appreciable scale. Consequently, research regarding neural circuit function is usually conducted in invertebrate model organisms like *Cancer borealis*, *Homerus americanus*, *Caenorhabditis elegans*, and *Drosophila melanogaster*. The gross anatomy of the invertebrate nervous systems resembles that of mammals, however, they are comprised of far fewer neurons, which are considerably more accessible than those in mice. Similarly, invertebrate and mammalian nervous systems share broad types of circuit [e.g., central pattern generators (CPGs)], neurons (e.g., sensory, higher, inter, and motor) and demonstrate conserved gene expression in certain cells (below). See Figure 1 for an overview, and “Comparison of *Drosophila* and Mammalian Neural Networks” for a more detailed comparison of the invertebrate (*Drosophila larval*) and human nervous systems.

While studies conducted in all of the invertebrate models listed above have made significant contributions to the field (White et al., 1986; Schulz et al., 2006, 2007; Daur et al., 2012; O’Leary et al., 2014), *Drosophila* offers distinct advantages over the others. Some of these are given in a published training package that serves as an excellent guide for researchers aspiring to work with the fruit fly (Roote and Prokop, 2013). Perhaps the most important advantages to mention explicitly here, are that flies are simpler to keep than crustaceans (no requirement for specialist equipment), they have thinner exoskeletons (easier access to neurons), and can be manipulated using the famous *Drosophila* genetic toolkit. Flies also possess a more complex nervous system [∼10,000 neurons in the larva (Heimbeck et al., 1999)] than the reductionist *C. elegans* [∼302 neurons (White et al., 1986)], making the former more relatable to mammals. Moreover, it has proven easier to perform electrophysiology on fly neurons than on those in the nematode worm. The latter lacks ganglionic structure and exhibits a high internal pressure that is problematic for dissection. This is significant, as electrophysiology plays an important role in characterising neurons by describing ionic and synaptic currents (Baines and Bate, 1998), and validating synaptic connectivity (Giachello et al., 2020). Finally, while the nematode worm is the only model organism to have an established and complete neural connectome (White et al., 1986), *Drosophila* is catching up quickly. This is especially true of the fruit fly *larval* connectome.

This review summarises recent progress made in characterising the *Drosophila* larval locomotor network. The summary is focused on the neurons and synapses that form a circuit associated with an identified premotor interneuron, A27h. It highlights similarities between the A27h-related circuit and those in mammals, to demonstrate the utility of the fly nervous system for studying the latter. Finally, it develops this point by discussing how the fly has been, and could be used, to explore critical periods of development and variability in neural circuits.

## The *Drosophila* Larval Connectome

Pioneering work published in 2015, produced a transmission electron microscopy volume of the entire nervous system of a female first-instar larva (Ohyama et al., 2015). This volume has since been used as the basis for reconstructions of neurons that describe their chemical synaptic connectivity, often with a focus on cells that contribute to the larval locomotor circuit. Table 1 lists these neurons and the publications that described them.

**TABLE 1:** List of Identified *Drosophila* Larval Neurons described by TEM Reconstructions.

Neuron(s)	Described by
A27h and GABAergic Dorsolateral Neurons (GDLs)	Fushiki et al. (2016)
A23a and A31k	Schneider-Mizell et al. (2016)
Excitatory interneurons 1-6 (eIN-1-6) and Inhibitory interneurons (iIN-1)	Zwart et al. (2016)
Cholinergic lateral interneurons 1 and 2 (CLI-1 and CLI-2)	Hasegawa et al. (2016)
Glutamatergic ventro-lateral interneurons (GVLIs)	Itakura et al. (2015)
Period-positive median segmental interneurons (PMSIs)	Kohsaka et al. (2014)
Saaghi-1-3,5 and even-skipped(+)	Heckscher et al. (2015)
Down-and-back 1 (dnb1)	Burgos et al. (2018)
Pair1 and Moonwalker Descending Neurons (MDNs)	Carreira-Rosario et al. (2018)
236 PMINs*	Zarin et al. (2019)

*Not all listed here for brevity.

In addition to the work summarized in Table 1, research has identified and partially characterised the thirty-three motor neurons that innervate the thirty body wall muscles of each larval hemisegment (Landgraf et al., 1997; Baines and Bate, 1998; Choi et al., 2004). It has also described six proprioceptors that contribute to crawling (Hughes and Thomas, 2007; Cheng et al., 2010; Vaadia et al., 2019). All of these MNs, and several proprioceptors, have been added to the connectome [(Zwart et al., 2016; Zarin et al., 2019) and (Heckscher et al., 2015; Schneider-Mizell et al., 2016), respectively]. Therefore, research has established a reasonably complete network of sensory, inter and motor neurons that form (part of) the larval locomotor circuit.

A complete discussion of every neuron implicated in the larval locomotor system is beyond the scope of this text. Instead, as mentioned in the Introduction, this review explores the locomotor network associated with one premotor interneuron, A27h, to demonstrate how fly circuits can be used to model mammalian circuits. The A27h-related circuit was chosen as an exemplar for two reasons. First, A27h is arguably the most completely described interneuron of the larval locomotor network. Second, it has already been used in work on critical periods of development (Giachello et al., 2019), which becomes relevant in the latter part of this text. This review, therefore, describes A27h and those neurons that are monosynaptically connected to it, with limited discussion of neurons that form polysynaptic connections to it.

### Summary of the *Drosophila* Larval Locomotor Circuit Associated With A27h

#### A27h and Its Role in Locomotion

*Drosophila* larvae are capable of numerous stereotyped behaviours, including nociceptive “rolling,” head casting, and forward or backward crawling. Crawling occurs as a result of the peristaltic contractions of ∼30 muscles (Landgraf et al., 1997) associated with body segments (Heckscher et al., 2012). Specifically, a wave of muscle contraction passes from the posterior to the anterior of the animal to move it forward (see Supplementary Animation 1), and in reverse to move it backward. Each complete wave, and so a single larval “stride,” is ∼1 s long. Muscle contractions are driven by a CPG of interneurons present in the animal’s ventral nerve cord [VNC, (Pulver et al., 2015), which is analogous to the mammalian spinal cord (Figure 1)]. Indeed, mammalian locomotion is generated by a very similar system (see below). Fictive rhythmic activity of the larval CPG persists in the absence of sensory input (Pulver et al., 2015), however, normal crawling is regulated by sensory information (Hughes and Thomas, 2007; Cheng et al., 2010). Goal-oriented locomotion (e.g., moving toward an olfactory attractant) occurs as a result of CPG-generated peristaltic waves, with direction changed when sensory input results in a course-correcting turn (Gomez-Marin and Louis, 2014). This type of CPG-led, motor program selection-influenced locomotion is also observed in mammals (see “Comparison of *Drosophila* and Mammalian Neural Networks”).

**FIGURE 2:**
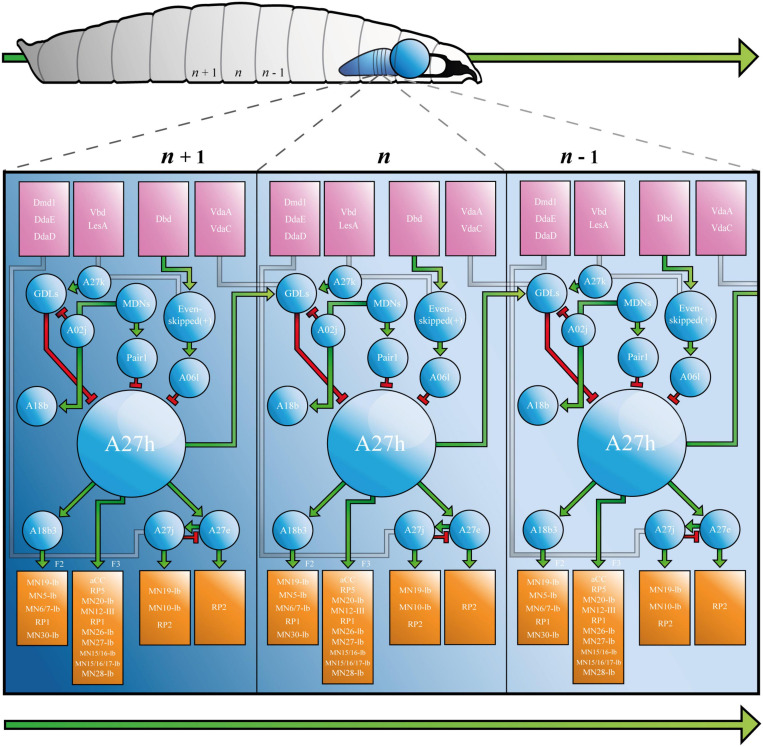
The *Drosophila* Larva A27h-related Locomotor Circuit. A, *Drosophila* larva showing ventral nerve cord (VNC) and brain lobe(s), with three segments labelled from most posterior (left, *n* - 1) to most anterior (*n* + 1, right). B, connectivity diagram of the neurons of the larval locomotor circuit. Sensory neurons appear in pink boxes, interneurons in blue circles and motor neurons in orange boxes. Small green arrows represent excitatory synapses, red T-bars represent inhibitory synapses, and grey paths show synapses that have not been completely characterised. F2 and F3 refer to muscle groups discussed in the text. Large green arrows shown in both A and B represent direction of travel, to illustrate that waves of neural activity move from posterior to anterior segments, as the larva crawls forward. See Supplementary Animation 1 for an animated version of this figure.

A27h is a cholinergic (excitatory) premotor interneuron that contributes to the larval CPG, which was originally identified by its synaptic connections (32–41 synapses detected in the left and right A1 hemisegments) to a population of GABAergic (inhibitory) dorsolateral interneurons [GDLs, (Fushiki et al., 2016)]. A27h arborises in the motor domain of the VNC and extends presynaptic terminals toward a specific motor neuron (MN), aCC (a.k.a. MN1-Ib) in the same segment (Figure 2). Indeed, dual electrophysiological recordings showed that current injection into A27h, depolarises aCC (Fushiki et al., 2016). More recent work employed tetrodotoxin-engineered resistance for probing synapses [TERPS, (Zhang and Gaudry, 2018)] to show that this occurs via a monosynaptic connection (Giachello et al., 2020). A27h also connects to the RP5 MN (MNISNb/d, Fushiki et al., 2016), and according to later reconstruction of all 236 premotor interneurons, to several others: MN-20Ib; MN12-III (V-MN); MN14Ib (RP1); MN26-Ib; MN27-Ib; MN15/16-Ib (MN-VO4/5); MN15/16/17-Ib (MN-VO4-6) and MN28-Ib (Zarin et al., 2019).

A27h acts via its myriad synapses, to contribute to forward, but not backward crawling (Fushiki et al., 2016). This observation, which was made by calcium imaging of the ventral nerve cord, has been refined to show that the premotor interneuron (PMIN) is most active during the contraction of a specific group of muscles (group “F3,” comprised of muscle 1, 8, 15, 16, 17, 20, and 28), which is associated with the late stages of segmental contraction during peristalsis (Zarin et al., 2019). Its activity also provides feed-forward inhibition of MNs in more anterior segments, via GDLs. A27h, therefore, contributes to forward crawling by promoting contraction of muscles in one segment, while simultaneously inhibiting the same muscles in the next (more anterior) segment (see Figure 2 and Supplementary Animation 1).

#### Neurons Downstream of A27h

A27h provides upstream monosynaptic input to several neurons besides those listed above. Specifically, reconstructions describe synaptic connections with interneurons A27e, A27j (Schneider-Mizell et al., 2016) and A18b3 [a.k.a. CLI-1 (Hasegawa et al., 2016)]. A27e is a PMIN that, according to Schneider-Mizzel et al., excites MN RP2 (MNISN). It is important to note that A27e is separate to a similarly named neuron that is also described in the connectome, A27e(2). The latter does not synapse with RP2, but with MN29-Ib, MN8-Ib (SBM) and MN22-23Ib (LT2/LT3) (Zarin et al., 2019). A27e has a reciprocal relationship with A27j (Schneider-Mizell et al., 2016); it excites GABAergic A27j, which, in turn, inhibits A27e (Figure 2). A27j also receives input from the sensory neurons dmd1, ddaD, and ddaE [proprioceptors described in Vaadia et al. (2019)] and synapses with RP2 (Schneider-Mizell et al., 2016; Zarin et al., 2019). Thus, A27j inhibits RP2 directly, and may also do so by reducing excitatory input from A27e.

A18b3 is a cholinergic premotor interneuron that is only active during forward crawling (Hasegawa et al., 2016; Zarin et al., 2019). Its activity therefore mirrors that of A27h, however, it synapses with different MNs. Hasegawa et al., used GFP reconstitution across synaptic partners (GRASP) to predict monosynaptic connections between A18b3 and aCC/RP2, but reconstructions performed by Zarin et al., only showed synapses between A18b3 and MN19-Ib, MN5-Ib (LO1), MN6/7-Ib (RP3), MN14-Ib (RP1), and MN30-Ib (RP4). Electrophysiology supports Zarin et al., as depolarising A18b3 does not change the membrane potential recorded in aCC (Giachello et al., 2020). This highlights the importance of using the same TERPS (electrophysiology) experiments that validated the connection between A27h and aCC (Giachello et al., 2020), to address controversy and validate synapses posed by TEM reconstruction(s). A18b3 signals (via relevant MNs) to coactive muscle group “F2” [muscles 3, 4, 5, 9, 12, 13, 18, 19, 25, 26, and 29 (Zarin et al., 2019)], which is (mostly) active earlier in segmental contraction than group F3. Thus, A27h drives contraction in one subset of muscles (F3), while simultaneously contributing to sustained activity in another (F2), via A18b3 (see Figure 2 and Supplementary Animation 1).

#### Neurons Upstream of A27h

The larval connectome predicts that A27h receives downstream monosynaptic input from GDLs (Fushiki et al., 2016), A06l [a.k.a. Saaghi-1 (Heckscher et al., 2015)] and Pair1 (Carreira-Rosario et al., 2018). GDLs were identified by screening for Gal4 lines expressed in GABAergic, rhythmically active neurons [i.e., those involved in the CPG (Fushiki et al., 2016)]. GDL activity occurs at approximately the same time as activity in aCC in the preceding segment, during both forward and backward locomotion. Indeed, GDLs appear to receive all of their inputs from adjacent segments. In addition to those from A27h, this includes inputs from sensory neurons vdaA and vdaC (see Figure 2). VdaA and vdaC are type II multidendritic (MD) neurons that respond to gentle touch (Tsubouchi et al., 2012). It therefore seems that GDLs integrate premotor and gentle touch-related signals, to promote relaxation of specific muscles during peristalsis. See Figure 2 and Supplementary Animation 1 for a depiction of the relationship between vdaA, vdaC, GDLs, A27h, and aCC in forward crawling. Finally, TEM data shows that GDLs do not form monosynaptic connections with MNs [(Fushiki et al., 2016) and personal communication with Aref Zarin], so must contribute to relaxation via premotor intermediaries.

The A06l IN is one of two neurons (A06l and A06e) that were identified by their presynaptic contacts with even-skipped(+) INs [a.k.a. A08e1-3, (Heckscher et al., 2015)]. Even-skipped(+) INs are unusual, because they do not demonstrate the rhythmic activity characteristic of many neurons associated with the A27h circuit. Indeed, they do not form part of the CPG. Rather, they receive input from dbd, vbd, and lesA sensory neurons and relay that information either to contralateral MNs, or via A06l and A06e to ipsilateral MNs (Heckscher et al., 2015). This pattern of projections, plus experiments that show thermogenetic manipulation of even-skipped(+) neurons leads to abnormal body posture, suggests that even-skipped(+) and so A06l and A06e INs, contribute to the symmetry of segmental muscle contraction. A06l and A06e are also connected to aCC (MN1-Ib), MN19-Ib, MN30-Ib (RP4) and others given in Zarin et al. (2019). See Figure 2 and Supplementary Animation 1 for a depiction of the role of A06l in the A27h-related locomotor circuit.

It is important that the research that identified A06l (Heckscher et al.), did not describe a connection between it and A27h. This synapse was described by Fushiki et al. (2016), who used a figure to present several connections between neurons, that were not discussed in the text. In addition to synapses between A06l and A27h, the authors showed that A27k [a.k.a Ifb-Bwd (Kohsaka et al., 2019)] and A02j [a “*period*-positive median segmental interneuron” (Kohsaka et al., 2014)] INs connect and provide inhibitory input to GDLs, implicating them in the A27h-related locomotor circuit (Figure 2). Reconstructions performed by Zarin et al., did not include the IN network associated with A06l. However, calcium imaging showed that A06l is rhythmically active during forward and backward peristalsis, at the same time as the inhibitory premotor IN A31k (Zarin et al., 2019). It could, therefore, form part of the A27h-associated CPG independent of its separate role downstream of even-skipped(+) INs. Consequently, A061 may be an example of a single neuron that can “switch” to contribute to more than one circuit (Gutierrez et al., 2013).

Pair1 cells are GABAergic INs subject to input from command-like (higher) moonwalker descending neurons [MDNs, (Carreira-Rosario et al., 2018)]. MDNs promote backward crawling in a process reminiscent of the motor program selection associated with regulation of CPGs in other animals, including mammals [below and (Grillner and El Manira, 2020)]. They signal Pair1 neurons to inhibit A27h, thereby ceasing forward crawling while simultaneously signaling a cholinergic IN, A18b, that contributes to backward crawling (Carreira-Rosario et al., 2018).

## Comparison of *Drosophila* and Mammalian Neural Networks

Following the overview of the larval connectome, it is important to establish that the fundamentals of the fly and mammalian locomotor systems are the same. Clearly, the gross anatomy of the two is similar (see Figure 1 and Introduction), and this similarity is retained at the higher level of resolution offered by considering the circuit-level components of each. The circuits that comprise the locomotor system of mammals have almost direct equivalents in larvae. Specifically, the striatum, basal ganglia (SNr) and globus pallidus interna (GPi) are responsible for locomotor program selection in mammals (Grillner and El Manira, 2020), as MDNs seem to be (at least in the case of Pair1 in backward crawling) in *Drosophila* (Carreira-Rosario et al., 2018). The mesencephalic locomotor region (MLR) provides locomotor command output in mammals (Grillner and El Manira, 2020), as Goro neurons do for rolling behaviour in larvae (Ohyama et al., 2015). Shox2+ interneurons that do not co-express Chx10, may generate rhythms in mammals (Dougherty et al., 2013) that are reminiscent of those observed in the A27h-related circuit in the fruit fly larva (above). Both drive muscle contraction through motor neurons that form motor pools (Landmesser, 1978; Landgraf et al., 1997) and muscle spindles provide proprioceptive feedback on mammalian muscle length during locomotion (Kroger and Watkins, 2021), in the same way that dorsal bipolar dendritic neurons may do during larval crawling (Hughes and Thomas, 2007; Suslak et al., 2015; Vaadia et al., 2019). The depth of circuit-level similarity that the field can draw will almost certainly grow, too, as the connectomes of the larva, and of mammalian models, are completed.

Similarities between the mammalian and *Drosophila* nervous systems extend beyond the circuit, to the molecular level. The transcriptional co-repressor protein, Groucho, mediates Class I and II homeodomain gene interactions necessary for normal development of motor neurons in mammals (Muhr et al., 2001), and performs a similar regulation of *even-skipped* expression in *Drosophila* (Jimenez et al., 1997; Kobayashi et al., 2001). LIM-HD and MNR2/Hb9 genes *islet-1*, *islet-2*, *Lhx3*, *Lhx4*, and *Hb9/MNR2* specify motor neurons in vertebrates (zebrafish and chicks), and their ortholog *islet*, *lim3*, and *Hb9* specify ventrally projecting MNs in flies [reviewed in Thor and Thomas (2002)]. *Bicoid* establishes the anterior-posterior axis required for the normal development of *Drosophila* embryos (Driever and Nussleinvolhard, 1988), and is the founder gene of a family that includes *Pitx2*. Expression of the latter defines a group of (V0_C_) interneurons that modulate murine locomotor activity (Zagoraiou et al., 2009). Similarly, mammalian *Dbx-1* specifies a group of V0 INs that are necessary for left-right hindlimb coordination in mice (Lanuza et al., 2004). Its *Drosophila* ortholog, *Dbx1/2*, is expressed in (mostly GABAergic) interneurons and is necessary for normal motor axon outgrowth (Lacin et al., 2009). There is clearly potential to map mouse and fly *dbx*-dependent neurons directly, by checking for *Dbx1/2* expression in cells identified in the larval connectome. Given that many of the neurons of the connectome are well characterised, doing so would provide insight into the function of *Dbx-1*-expressing V0 INs, and so exemplifies how larval circuits could help decode the more complex mammalian network. Finally, murine V1 interneurons that transiently express the transcription factor, *Engrailed 1 (En1)*, differentiate into inhibitory interneurons that synapse with motor neurons (Sapir et al., 2004). Persistent expression of the *Drosophila* ortholog of *En1*, *Engrailed*, is necessary for normal sensory axon trajectory, branching and target recognition (Marie et al., 2002).

Despite the long list of similarities between the *Drosophila* and mammalian locomotor systems, whether or not human INs form CPGs is a matter of debate (Minassian et al., 2017). It is, however, logical that CPGs are phylogenetically conserved. In addition to the evidence for CPGs in mammalian models (some of which is described to above), research on spinal cord injury (SCI) patients makes a convincing case for locomotor CPGs in humans (reviewed in Guertin, 2013). For example, SCI patients produce spontaneous, rhythmic, involuntary leg movements (Bussel et al., 1988; Calancie, 2006) and applying an epideural stimulation to their lumbar spinal cord, produces involuntary rhythmic flexion-extension movement of the legs (Dimitrijevic et al., 1998). Assuming they exist, understanding human locomotor CPGs could lead to treatments for myoclonus (involuntary leg movements symptomatic of SCI), multiple sclerosis, restless leg syndrome (RLS) and alternating leg muscle activation (Yokota et al., 1991; Chervin et al., 2003; Tassinari et al., 2005, 2009; Cosentino et al., 2006; Schurks and Bussfeld, 2013). Again, the complexity and ethical limitations of studying CPGs in humans/mammals, means that *Drosophila* offers a more tractable system in which to progress this understanding. This is especially true for RLS, as it can be modelled in the fly through mutation of an ortholog of a risk factor gene, BTBD9 (Freeman et al., 2012). An investigation of the circuit and molecular-level implications of BTBD9 mutation on the larval locomotor circuit, might provide insight into the mechanisms of RLS that would be difficult to achieve in any other animal, in the same timeframe.

Finally, while a direct comparison of the *Drosophila* and mammalian (and particularly human) locomotor systems is helpful, it should not limit the potential application of the larval circuit in research. The larval locomotor circuit can, and should, be used to understand more general principles of neural circuit development and function. These principles may apply to important subjects in neuroscience, such as critical periods of development, variability in neural circuits, or almost any other circuit or mechanism-related subject related to neural networks. With that in mind, the next section of this review explores critical periods of development and variability.

## Using the A27h-Related Larval Locomotor Circuit to Model Critical Periods of Neural Development

Critical periods (CPs) are defined windows of developmental time that are characterised by high levels of neural plasticity (Hensch, 2005; Reh et al., 2020). This plasticity facilitates fine-tuning of neural networks according to external (environmental) and internal (genetically determined and activity-dependent) cues. It has been posed that this tuning includes encoding fixed “set points” of activity that are required for homeostatic mechanisms to function in the mature neuron or network (Giachello and Baines, 2015). It is possible that aberrant activity during CPs causes “set points” to be fixed outside of physiologically normal ranges. This may, in turn, lead to an unstable network prone to hypo- or hyperactivity in the adult. Neural hyperactivity is associated with seizure (Scharfman et al., 2008) and so, perhaps unsurprisingly, aberrant activity during critical periods of development has been linked to epilepsy, autism and schizophrenia (Rice and Barone, 2000).

CPs are conserved across phyla, and a number of them have been identified in humans (Levitin and Zatorre, 2003) and other mammals (Hubel and Wiesel, 1970; Kirkwood and Bear, 1994; Yamaguchi and Mori, 2005; Tanaka et al., 2009). Humans and mammalian models have facilitated research that shows it is possible to correct aberrant activity during CPs to prevent seizure (Blumenfeld et al., 2008; Marguet et al., 2015). They have also been used to demonstrate that CPs can be reopened (Maya-Vetencourt et al., 2008), so that symptoms of dysfunction can be treated in later life (Hensch and Bilimoria, 2012; Marguet et al., 2015). Experiments in humans and mammalian models are, however, constrained by the limitations described in the introduction. CPs have therefore been identified and interrogated in a number of models more conducive to experimentation: *Danio rario* (Riley et al., 1997; Moorman et al., 2002; Avitan et al., 2017; Xie et al., 2019); *H. americanus* (Govind and Pearce, 1989); *C. elegans* (Swanson and Riddle, 1981), and *Drosophila* (Fushiki et al., 2013; Marley and Baines, 2011; Giachello and Baines, 2015). The identification of CPs in the fruit fly means that the A27h-related larval locomotor circuit provides an exciting opportunity to provide network, cellular and mechanism-level resolution insight into the role of CPs in neurodevelopment.

**FIGURE 3:**
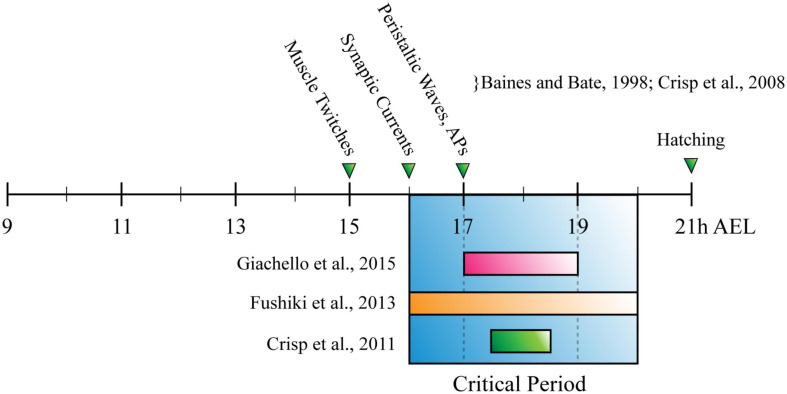
The *Drosophila* Larva Critical Period for Locomotor Development. Timeline for *Drosophila* embryonic development, measured in hours after egg laying (h AEL). Research has described the critical period for locomotor development (blue box) as being: (1) 17–19h, through manipulation of cholinergic neuron activity (pink bar); (2) 2 h between 16–20 h AEL, through manipulation of chordotonal neuron input to the nervous system (orange bar); (3) 90–30 min before tracheal filling, or ∼17 h 30 min–18 h 30 min AEL (green bar). These timings overlap with each other, occurring shortly after the appearance of myogenic movements (muscle twitches) and around the same time as the emergence of synaptic currents and peristaltic waves.

The CP for locomotor network development described in *Drosophila*, was established by showing that exposing *slamdance* (seizure) mutants to phenytoin (a commonly used antiepileptic) during just embryogenesis, is sufficient to prevent seizure-like activity that otherwise occurs in third-instar larvae (Marley and Baines, 2011). Later work from the same group reported a similar outcome after manipulating activity during the CP, by altering temperature or administering picrotoxin [a proconvulsant/GABA_A_ inhibitor (Giachello and Baines, 2015)]. It also employed optogenetics to refine the critical period to 17–19h after egg laying [(AEL), 80–90% embryonic development (Giachello and Baines, 2015)]. Interestingly, this period corresponds with both the emergence of patterned peristaltic contractions of body wall muscles in the developing embryo [∼17 h AEL, (Baines and Bate, 1998; Crisp et al., 2008)], and CPs for all sensory (Crisp et al., 2011) and chordotonal neuron-specific input into the larval locomotor circuit [(Fushiki et al., 2013), see Figure 3]. This convergence is consistent with different neurons of the larval locomotor circuit (and perhaps the whole NS) undergoing significant, activity-dependent fine-tuning, simultaneously. Similarly, the fact that development of normal crawling requires activity in two specific, entirely separate populations of neurons, opposes the popular idea of widespread degeneracy in neural circuits (Leonardo, 2005; Cropper et al., 2016; Marder et al., 2017). The effect of activity manipulation during the CP was prevented by prior exposure to anticonvulsant drugs or optogenetics (Giachello and Baines, 2015). Thus, experiments conducted in *Drosophila* agree with those conducted in mammals. Both show it is possible to prevent symptoms associated with disorders of neurodevelopment, by correcting aberrant activity during a CP.

Recent work by Giachello et al. exploited the unique genetic tractability and accessibility of the A27h-related locomotor circuit, to investigate the activity of defined populations of neurons during the CP (Giachello et al., 2019). Specifically, disturbing activity in A27h-Gal4-expressing neurons (∼6 per segment) during embryogenesis, was sufficient to replicate the effect observed when activity was disturbed across the whole nervous system. This important result suggests that some neurons may make larger contributions to setting physiologically relevant network parameters (e.g., homeostatic set points) than others. It could also be argued that regardless of those parameters, A27h is a particularly important neuron in the locomotor circuit. It is possible that it acts a network “oscillator” that generates bursts of APs to drive circuit rhythms (Marder et al., 2015). If specific neurons do make larger contributions to development than others and this is conserved in mammals, “keystone” cells could be targeted with gene therapy, or drugs administered once a CP has been reopened (Maya-Vetencourt et al., 2008; Gervain et al., 2013), to help treat or possibly cure disorders of neurodevelopment (Hensch and Bilimoria, 2012). This highlights the impact that investigating CPs in *Drosophila* larvae could have, and future studies should ask: “which are the mechanisms that define critical periods of development?,” and: “what is the safest and most effective method for correcting aberrant activity during critical periods, to prevent disorder occurring?”

Finally, work that was published while this review was written, used the *Drosophila* larval locomotor circuit to address the first of the two aforementioned questions. Specifically, the authors used an optogenetic protocol reminiscent of that employed by Giachello and Baines (2015), to show that changes in activity during the CP regulate MN dendrite length, complexity and connectivity (Ackerman et al., 2021). The authors also showed that astrocyte to MN signaling closes the CP. This work is, therefore, a very recent demonstration of the application of work in fly circuits, to further our understanding of mechanisms that likely impact the development and function of all nervous systems.

## Using the A27h-Related Larval Locomotor Network to Model Circuit Variability

For the purposes of this review, variability describes interindividual differences in neural circuits. Variability may occur as a result of different genotypes, for instance, due to genetic mutations (Huguet et al., 2016) that could explain the excessive variability in neural activity observed in autism (Dinstein et al., 2012; Weinger et al., 2014; Haigh et al., 2016). It might also occur in animals with identical genotypes due to their environment, as it does in crabs exposed to different temperatures (Alonso and Marder, 2020), and in random processes of development such as noisy filopodial outgrowth (Lan and Papoian, 2008). Variability manifests as differences in anatomy (Daur et al., 2012) or activity (Giachello and Baines, 2015; Giachello et al., 2019), and is usually tolerated by homeostasis or actively utilised in development. Normal levels of variability are advantageous. They are linked to learning (Olveczky et al., 2011; Hedden and Gabrieli, 2004) and robustness in both individuals and populations of animals, with the latter due to “evolutionary innovation” developed through tolerance of mutation (Hiesinger and Hassan, 2018). In contrast, excessive variability is detrimental to an animal’s fitness (Dinstein et al., 2015) and may reduce the effectiveness of activity manipulation as a treatment or cure for neurological disorders. It is, therefore, important to study variability and its impact on neural networks.

Research performed in humans has produced some notable results regarding variability in neural circuits. There are significant differences in the sulcal and gyral patterns of brains of monozygotic twins (Mohr et al., 2004), and imaging embryos reveals asymmetry in the number and length of branches between the left and right ulnar and radial nerves (Belle et al., 2017). This work is, however, (again) limited by the complexity and inaccessibility of the human NS. Much of what is known about variability has, therefore, been gleaned from work on identified neurons in invertebrates. For example, research has shown that developmental cell competition results in stochastic survival of neurons in *C. elegans* (White et al., 1986), and has demonstrated variability in the circuits of crustacea [reviewed in Marder et al. (2015)]. This includes differences in anatomy that produce reliable behaviour(s): gut constrictor muscle p2 of *H. americanus* is innervated by 3-7 plyoric neurons (Bucher et al., 2007), but excitatory post-synaptic potentials measured in p2 are identical regardless of the number of (pyloric) neurons that synapse with it (Daur et al., 2012). Similarly, intracellular dye fills show animal-to-animal variation in soma position and branching pattern of Anterior Gastric Receptor neurons in the stomatogastric ganglion (STG) of *C. borealis* (Goeritz et al., 2013). The physiology of other STG neurons (Gastric Mill neurons) is maintained despite this variability, and the “sloppy” tuning that compensates for it (Otopalik et al., 2017).

Research in *Drosophila* shows variability across the fly nervous system. It reports interindividual differences in the number of ommatidia per compound eye, despite the precise wiring of photoreceptor axons (Vollmer et al., 2016). Noisy, cell autonomous expression of Down syndrome cell adhesion molecule (*Dscam*) facilitates self-avoidance in filopodial outgrowth (Matthews et al., 2007; Zipursky and Grueber, 2013) through alternative splicing that generates ≤ 19,008 protein isoforms, which contribute to (self) recognition (Schmucker et al., 2000). Noisy wiring contributes to proper development of the fly olfactory system (Tobin et al., 2017), and flies demonstrate interindividual differences in “handedness,” which describes individual directional preference (probability of left or right-turn decisions) in animals navigating a maze (Buchanan et al., 2015). The *Drosophila* NS overcomes stochastic expression of ion channel genes *Shal* and *Shaker* through reciprocal regulation, which ensures normal *I*_*A*_ current is maintained over time (Bergquist et al., 2010) and of course, exhibits variable behaviour following perturbation of activity during critical periods of development (Fushiki et al., 2013; Marley and Baines, 2011; Giachello and Baines, 2015; Giachello et al., 2019). Thus, invertebrate, and especially crustacean and fly model networks, have been used extensively to investigate variability, and demonstrate the power in doing so in simple circuits.

The A27h-related locomotor circuit presents an ideal opportunity to address some of the many remaining, interesting questions that the field must answer regarding variability. These include: “how much of a healthy nervous system is variable, and how much is fixed?,” plus: “what is the threshold that defines advantageous versus deleterious variability?” It is very well suited to doing so because of the generic advantages *Drosophila* provides over other model organisms (Roote and Prokop, 2013), and for several other reasons. The first is that A27h is part of an established connectome of identified neurons (see above) that is reliable enough to use to study variability. This reliability facilitates straightforward manipulation of (variable) parameters *in vivo* (as in Giachello and Baines, 2015), and makes accurate *in silico* modelling of the larval locomotor network relatively simple. This is important, as models play a key role in predicting behaviour in neural networks (Prinz et al., 2004; O’Leary et al., 2014; Alonso and Marder, 2020). Moreover, technological advances focused on accelerating acquisition of *Drosophila* connectome image volumes, such as Gridtape (Graham et al., 2020) and the FlyEM project (Janelia Research Campus), could provide unique insight into variability in connectivity. For example, comparison of circuits that developed with and without activity perturbation during CPs (Giachello and Baines, 2015; Giachello et al., 2019), might provide insight into whether differences in activity alter connectivity. The fly larval locomotor circuit therefore offers a unique opportunity to progress basic research, and to elucidate causes of human disorders such as autism, schizophrenia, ADHD, dyslexia and epilepsy, that may be linked to variability in circuit structure, due in turn, to altered activity during CPs of development (Dinstein et al., 2015).

## Conclusion

While the ultimate goal is to map the whole *Drosophila* larval connectome, the field has already described several relatively complete larval circuits [see (Clark et al., 2018), for a summary], including the A27h-related locomotor circuit. Specifically, a combination of reconstructions based on a TEM volume (Ohyama et al., 2015) has posed connections between A27h and identified motor (Landgraf et al., 1997; Baines and Bate, 1998; Choi et al., 2004), inter (Kohsaka et al., 2014; Heckscher et al., 2015; Itakura et al., 2015; Fushiki et al., 2016; Hasegawa et al., 2016; Schneider-Mizell et al., 2016; Yoshikawa et al., 2016; Zwart et al., 2016; Burgos et al., 2018; Carreira-Rosario et al., 2018; Zarin et al., 2019), higher (Carreira-Rosario et al., 2018) and sensory neurons (Hughes and Thomas, 2007; Cheng et al., 2010; Fushiki et al., 2013; Vaadia et al., 2019). Some of these synapses have been validated by electrophysiology (Giachello et al., 2020) and so provide the basis for a reliable circuit that can be used to model others. Many of the neurons in this circuit can be manipulated using the *Drosophila* genetic toolkit, in conjunction with optogenetics and electrophysiology [as in Giachello et al. (2019)]. They therefore provide a degree of experimental utility that is rare, and that can be exploited to answer some of neuroscience’s most pressing questions. Those might be broad, such as: “how do neural circuits generate behavior?” or related to subjects like CPs, or variability in neural circuits. Regardless of the question, the high degree of conservation across species means that whatever is learned by answering it in *Drosophila*, will likely translate to humans.

## Author Contributions

IH wrote the manuscript and prepared the figure and animation. BC and RB provided significant feedback on, and made revisions to several drafts. AZ proof-read and made corrections to the final draft of the manuscript. All authors read and approved the submitted version.

## Conflict of Interest

The authors declare that the research was conducted in the absence of any commercial or financial relationships that could be construed as a potential conflict of interest.

## Supplementary Material

Supplementary Animation 1 Activity in the *Drosophila* Larva A27 h-related Locomotor Circuit. Animation is a copy of Figure 2, with depolarised neurons highlighted in green, and hyperpolarised neurons highlighted in red. Activity is depicted as it is described in the text, and is shown progressing from posterior (*n* + *1*) to more anterior segments (*n*, *n - 1*) of the body and ventral nerve cord. The progress of this activity has been slowed down (4 s per segment in animation, versus ∼1 s per stride in real time) to improve clarity.
